# Sclerostin antibody corrects periodontal disease in type 2 diabetic mice

**DOI:** 10.1172/jci.insight.181940

**Published:** 2024-07-18

**Authors:** Hakan Turkkahraman, Shannan Flanagan, Tianli Zhu, Nisreen Akel, Silvia Marino, Dayane Ortega-Gonzalez, Xue Yuan, Teresita Bellido

**Affiliations:** 1 Department of Orthodontics and Oral Facial Genetics, Indiana University School of Dentistry, Indianapolis, Indiana, USA.; 2 Department of Otolaryngology–Head and Neck Surgery, Indiana University School of Medicine, Indianapolis, Indiana, USA.; 3 Department of Biomedical Sciences and Comprehensive Care, Indiana University School of Dentistry, Indianapolis, Indiana, USA.; 4 Department of Physiology and Cell Biology, University of Arkansas for Medical Sciences, Little Rock, Arkansas, USA.; 5 Indiana Center for Musculoskeletal Health, Indiana University School of Medicine, Indianapolis, Indiana, USA.; 6 Central Arkansas Veterans Healthcare System, John L. McClellan, Little Rock, Arkansas, USA.

**Keywords:** Bone biology, Inflammation, Bone disease, Diabetes, Mouse models

## Abstract

Type 2 diabetes (T2D) is on the rise worldwide and is associated with various complications in the oral cavity. Using an adult-onset diabetes preclinical model, we demonstrated profound periodontal alterations in T2D mice, including inflamed gingiva, disintegrated periodontal ligaments (PDLs), marked alveolar bone loss, and unbalanced bone remodeling due to decreased formation and increased resorption. Notably, we observed elevated levels of the Wnt signaling inhibitor sclerostin in the alveolar bone of T2D mice. Motivated by these findings, we investigated whether a sclerostin-neutralizing antibody (Scl-Ab) could rescue the compromised periodontium in T2D mice. Administering Scl-Ab subcutaneously once a week for 4 weeks, starting 4 weeks after T2D induction, led to substantial increases in bone mass. This effect was attributed to the inhibition of osteoclasts and promotion of osteoblasts in both control and T2D mice, effectively reversing the bone loss caused by T2D. Furthermore, Scl-Ab stimulated PDL cell proliferation, partially restored the PDL fibers, and mitigated inflammation in the periodontium. Our study thus established a T2D-induced periodontitis mouse model characterized by inflammation and tissue degeneration. Scl-Ab emerged as a promising intervention to counteract the detrimental effects of T2D on the periodontium, exhibiting limited side effects on other craniofacial hard tissues.

## Abstract 2

A sclerostin-neutralizing antibody reverses bone loss, mitigates inflammation, and partially restores periodontal ligaments in a preclinical model of type 2 diabetes-induced periodontal disease.

## Introduction

The alveolar bone is a specialized segment of bone within the maxillary and mandibular regions that encapsulates and provides support to the roots of teeth. Its role is to ensure that teeth are securely anchored, thereby effectively distributing the forces generated during mastication (1, 2). Continuous remodeling of alveolar bone, driven by balanced osteoblast and osteoclast activity regulated through biochemical signaling, mechanical signals, and other cues, is essential for maintenance of the tooth-bone interface and adaptation to changing demands (3). Disruptions that cause a loss of stable alveolar bone support can lead to loosening of tooth roots, an increased risk of infection, and ultimately tooth loss and compromised masticatory function (1–3).

Type 2 diabetes (T2D), the most common form of diabetes, is rapidly surging on a global scale and is known to be linked with complications in the oral cavity, including gingivitis and periodontitis (4, 5). While gingivitis can be reversed, periodontitis damages the soft tissue and alveolar bone permanently, causing tooth mobility and, subsequently, tooth loss. Therefore, there is an urgent need to develop therapeutic interventions capable of halting this irreversible cascade of events in periodontitis.

Sclerostin, primarily secreted by osteocytes, inhibits bone formation by antagonizing the Wnt signaling pathway (6). Patients with genetic conditions characterized by inherited sclerostin deficiency exhibit high bone mass and a reduced risk of fractures (7, 8). In 2019, the FDA approved Evenity (romosozumab), a neutralizing anti-sclerostin antibody (Scl-Ab), for treating osteoporosis in postmenopausal women at high risk of bone fracture (9). Prior research has demonstrated that bones from type 1 diabetic mice exhibit increased expression of sclerostin in osteocytes (10) and that mRNA expression of *Sost*, the gene encoding sclerostin, is higher in diabetic bones compared with nondiabetic ones (11). Although we did not detect changes in *Sost* expression in bone or circulating sclerostin in our recent study in T2D mice, transcriptome analysis demonstrated a profound decrease in Wnt signaling (12). Furthermore, the restoration of bone mass, bone formation rate, and strength induced by anabolic ligands of the parathyroid hormone 1 receptor teriparatide and abaloparatide (ABL) in T2D mice to control levels was accompanied by a reduction in *Sost* expression in bone, and ABL also reduced sclerostin levels in the circulation (12). It is established that, in rodents, inhibition of sclerostin promotes osteoprogenitor proliferation and their recruitment to active bone surfaces, resulting in increased osteoblast number and activity (13). Scl-Ab was also shown to reactivate quiescent bone-lining cells that reside on inactive bone surfaces and convert them into active osteoblasts (14). In addition, Scl-Ab inhibits bone resorption through activation of Wnt signaling and increased expression of the Wnt target gene osteoprotegerin (15). Taken together, these findings raised the possibility that Scl-Ab would be beneficial in the context of diabetes. In fact, our recent study provided evidence that treatment with Scl-Ab reversed the bone loss induced by T2D in mice and corrected the changes in the diabetic bone signature at the tissue, cell, and molecular levels in vertebral and long bones (12). Furthermore, previous studies showed that Scl-Ab reversed the low bone mass and delayed bone healing observed in diabetic Zucker diabetic fatty (ZDF) rats (16).

Heretofore, the preclinical and clinical studies with Scl-Ab have focused on the effects of the therapy on the bones of the axial and appendicular skeleton. In contrast, the effect of Scl-Ab on craniofacial bones or the oral cavity remained to be defined, thereby precluding its application to oral complications of diabetes. In the current study, we investigated the effects of Scl-Ab on craniofacial tissues in healthy and T2D mice. We demonstrate that mice with nongenetic adult-onset T2D developed severe alveolar bone loss and periodontal soft tissue degeneration, mimicking the periodontal disease exhibited by patients with T2D. Moreover, treating T2D mice with Scl-Ab increased alveolar bone volume, reversed the effects of T2D on remodeling by increasing osteogenic cells and decreasing osteoclasts, and completely rescued the bone loss. Remarkably, the Scl-Ab treatment elicited protective effects on the periodontal soft tissues as well, by promoting proliferation of disintegrated periodontal ligament (PDL) cells, increasing collagen fiber density, preserving extracellular matrix expression, and reducing inflammation. Despite the robust bone-anabolic response of the alveolar bone to Scl-Ab, other craniofacial sites were only minimally affected, with mild bone accretion occurring in the edentulous and mandibular bones and a minor effect on the premaxillary suture. These findings demonstrate the therapeutic potential of treatment with Scl-Ab to restore alveolar bone and reverse the periodontal disease induced by T2D.

## Results

### T2D mice displayed elevated gingival inflammation and PDL degeneration.

**Figure 1:**
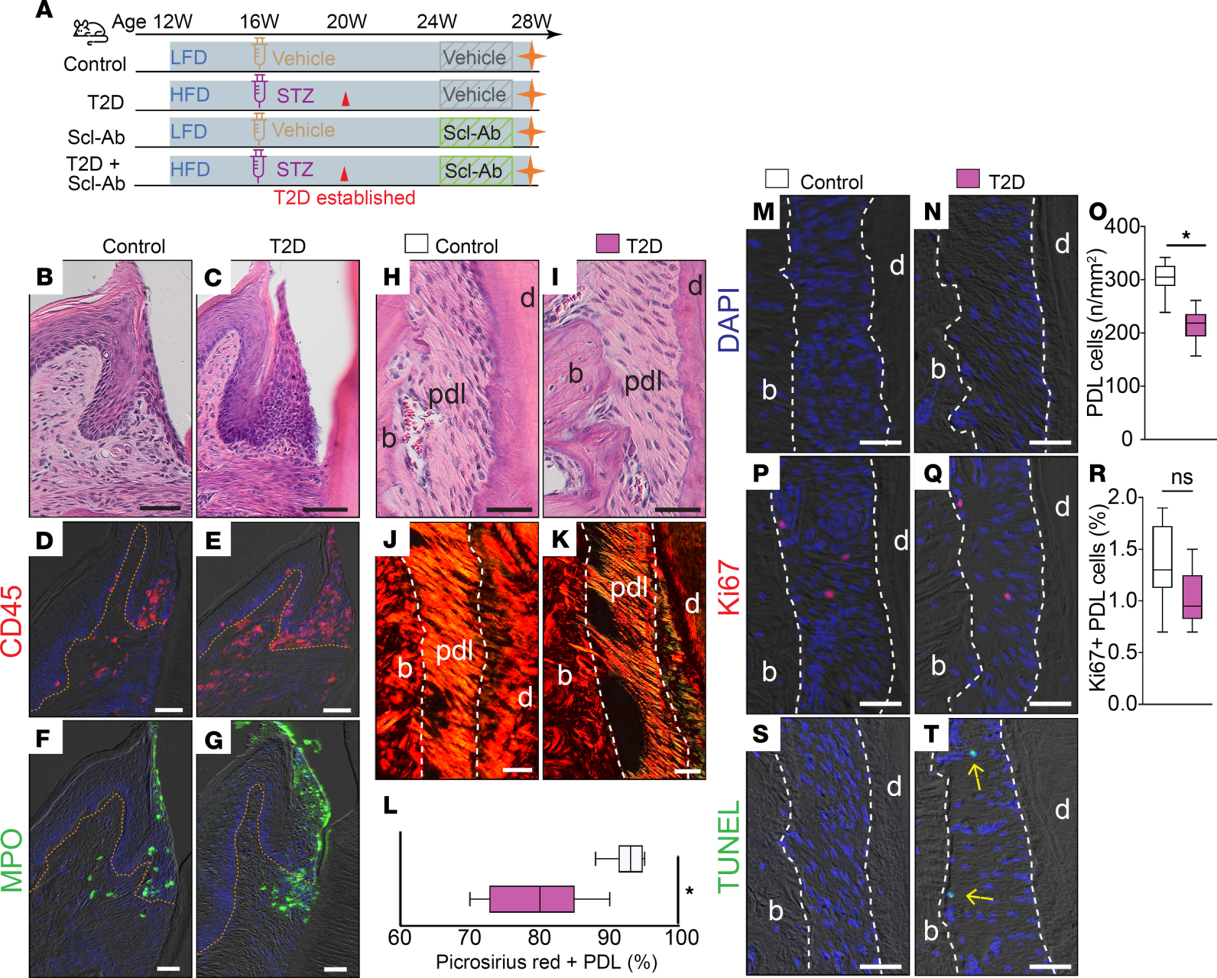
T2D mice displayed elevated gingival inflammation and PDL degeneration. (**A**) Schematic of the study design. (**B** and **C**) H&E staining shows the gingiva. (**D** and **E**) Immunostaining of CD45. (**F** and **G**) Immunostaining of the neutrophil marker myeloperoxidase (MPO). The orange dotted lines indicate the boundary between the epithelium and the underlying lamina propria. (**H** and **I**) H&E staining shows the PDL. (**J** and **K**) Picrosirius red staining, viewed under polarized light. (**L**) Quantification of the Picrosirius red–positive PDL area (*n* = 8). *P < 0.01. (**M** and **N**) DAPI staining shows cells in the PDL. (**O**) Quantification of PDL cells from DAPI-stained slides (*n* = 8). *P < 0.001. (**P** and **Q**) Immunostaining of Ki67. (**R**) Quantification of Ki67^+^ PDL cells (*n* = 8). ns, not significant. (**S** and **T**) TUNEL staining shows cell apoptosis in the PDL. Yellow arrows indicate TUNEL^+^ apoptotic PDL cells. The white dashed lines indicate the boundary of PDL. The data were analyzed using the Student’s *t* test. Scale bar: 50 μm. LFD, low-fat diet; HFD, high-fat diet; STZ, streptozotocin; T2D, type 2 diabetes; Scl-Ab, sclerostin antibody; pdl, periodontal ligament; b, bone; d, dentin.

Young adult mice were randomized into 2 groups (Figure 1A, first 2 groups): a control group fed a low-fat diet (LFD) and an experimental group fed a high-fat diet (HFD). After 4 weeks on the HFD, the experimental group received streptozotocin (STZ) injections to induce T2D, which was confirmed by non-fasting blood glucose measured 4 weeks later. All mice were maintained on their respective diets for an additional 4 weeks and treated with either a vehicle or Scl-Ab for another 4 weeks (Figure 1A). At the end of the experiment, mice with established T2D administered with a vehicle exhibited signs of gingival inflammation characterized by lymphocyte infiltration (Figure 1, B and C), increased CD45^+^ immune cell accumulation (Figure 1, D and E), and an elevated number of neutrophils compared with that in control mice (Figure 1, F and G). In addition to the inflamed gingiva, the PDL in T2D mice displayed reduced collagen fiber density (Figure 1, H–L) and decreased cell density (Figure 1, M–O). Although mitotic activity was low in both control and T2D mice (Figure 1, P–R), the PDL of T2D mice had more TUNEL^+^ cells, indicating increased apoptosis (Figure 1, S and T). These data suggest degeneration of the PDL linked to increased fibroblast apoptosis in T2D mice compared with control mice, in which cell apoptosis was rare.

### T2D mice displayed alveolar bone loss with elevated sclerostin expression.

**Figure 2:**
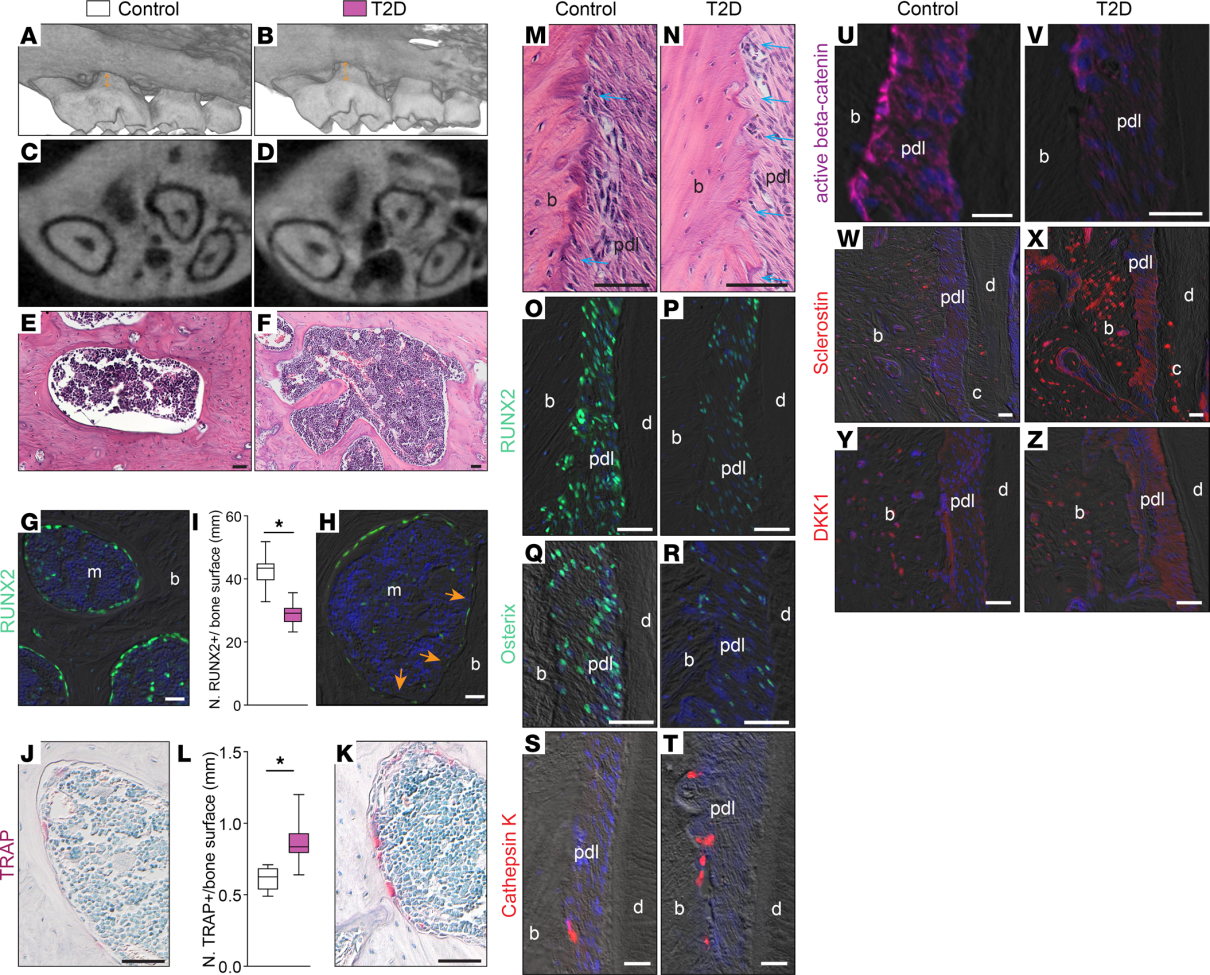
T2D mice displayed alveolar bone loss with elevated sclerostin expression. Representative 3D renderings of micro-CT scanning of (**A**) control and (**B**) T2D maxilla. Arrows indicate the distance from the cementum-enamel junction to the alveolar bone crest. (**C** and **D**) Representative 2D micro-CT transverse sections through the alveolar bone around the maxillary first molars. (**E** and **F**) H&E staining shows the periodontium in the control and T2D groups. (**G** and **H**) Immunostaining of RUNX2. Arrows indicate the bone surface that lacks RUNX2^+^ cells. (**I**) Quantification of RUNX2^+^ cells lining the bone surface (*n* = 8). *P < 0.001. (**J** and **K**) TRAP staining shows bone resorption activity. (**L**) Quantification of TRAP^+^ cells on the bone surface (*n* = 8). *P < 0.01. (**M** and **N**) H&E staining shows the alveolar bone on the mesial side. Arrows indicate the eroded bone surface. Immunostaining of (**O** and **P**) RUNX2, (**Q** and **R**) osterix, (**S** and **T**) cathepsin K, (**U** and **V**) active β-catenin, (**W** and **X**) sclerostin, and (**Y** and **Z**) DKK1. The data were analyzed using the Student’s *t* test. Scale bar: 50 μm. pdl, periodontal ligament; b, bone; m, marrow; d, dentin; c, cementum.

We further evaluated the alveolar bone using micro-CT and found a decrease in alveolar bone height, along with substantial interradicular alveolar bone loss (Figure 2, A–D). This was substantiated by a microscopic examination of the maxillary bones (Figure 2, E and F). RUNX2^+^ osteogenic cells, potentially osteoblasts or osteoprogenitors, were reduced on the bone surface of T2D maxillary bone compared with that in controls (Figure 2, G–I). In contrast, osteoclast numbers were higher in T2D maxillary bone compared with those in controls (Figure 2, J–L).

Examination of the PDL regions demonstrated an increase in eroded bone surface on the mesial side in the T2D group (Figure 2, M and N). Using RUNX2 and osterix as markers for the osteoblast lineage and cathepsin K as a marker for osteoclasts, we found that T2D PDLs exhibited a reduction in the number of osteogenic cells (Figure 2, O–R) and an increase in the number of osteoclasts (Figure 2, S and T). Notably, T2D mice had fewer active β-catenin^+^ cells on the bone surface, indicating impaired Wnt signaling (Figure 2, U and V).

We examined the expression patterns of 2 Wnt inhibitors, sclerostin and Dickkopf-1 (DKK1). In control mice, sclerostin was expressed by osteocytes (Figure 2W). T2D mice displayed strong sclerostin expression around osteocytes in alveolar bone, with some PDL areas also positive (Figure 2X). Similarly, DKK1 expression was upregulated in T2D mice (Figure 2, Y and Z). These results demonstrate excessive Wnt inhibitor production by osteocytes in T2D mice, which may disrupt bone remodeling balance, favoring resorption.

### Scl-Ab treatment completely reverses T2D-induced alveolar bone loss.

**Figure 3:**
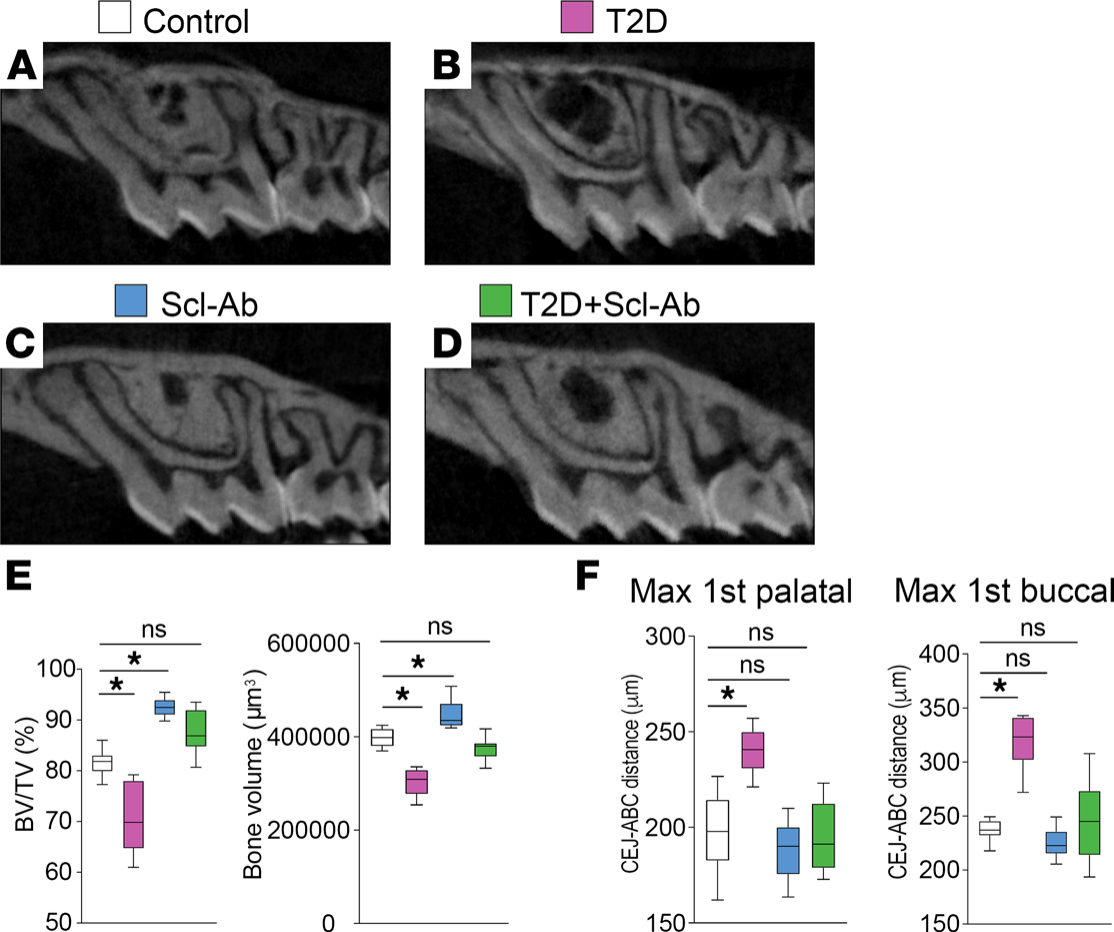
Scl-Ab treatment completely reverses T2D-induced alveolar bone loss. (**A**–**D**) Representative 2D micro-CT sections through the alveolar bone around the maxillary first molars. (**E**) Quantification of bone volume (BV) and the ratio of BV to tissue volume (TV) (*n* = 8). *P < 0.01; ns, not significant. (**F**) Quantification of the distance from CEJ to ABC (*n* = 8). *P < 0.001. The data were analyzed using 1-way ANOVA with Tukey’s post hoc tests. Max 1st, maxillary first molar.

To investigate whether suppressing excessive sclerostin could reverse the detrimental periodontal effects of T2D, we administered Scl-Ab to T2D and control mice (Figure 1A). Scl-Ab treatment increased maxillary bone volume in control mice and completely reversed the decrease in alveolar bone volume induced by T2D (Figure 3, A–E, and Supplemental Figure 1, A–E; supplemental material available online with this article; https://doi.org/10.1172/jci.insight.181940DS1). Moreover, the basal bone, which provides structural support to the teeth, became thinner in the T2D mice (Supplemental Figure 1, F and G). However, Scl-Ab treatment substantially thickened it in both control and T2D mice (Supplemental Figure 1, H–J). We also measured the cementum-enamel junction to alveolar bone crest (CEJ-ABC) distance on both the palatal and buccal sides. The increased CEJ-ABC distance observed in the T2D group indicated alveolar bone height loss, which was entirely rescued by Scl-Ab treatment (Figure 3F).

### Scl-Ab treatment promotes robust alveolar bone formation.

**Figure 4:**
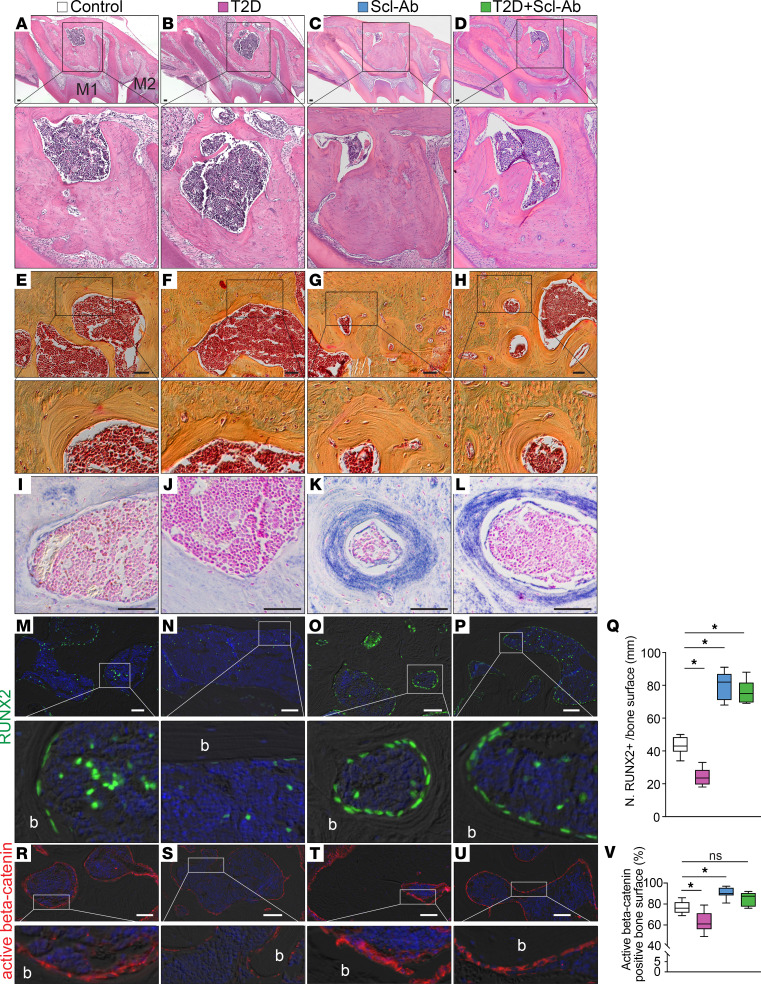
Scl-Ab treatment promotes robust bone formation. (**A**–**D**) H&E staining shows the alveolar bone under the furcation area. (**E**–**H**) Pentachrome staining shows the bone around the marrow cavity. (**I**–**L**) ALP staining shows bone formation activity around the marrow cavity. (**M**–**P**) Immunostaining of RUNX2. (**Q**) Quantification of RUNX2^+^ bone-lining cells on the bone surface (*n* = 8). *P < 0.0001. (**R**–**U**) Immunostaining of active β-catenin. (**V**) Quantification of active β-catenin–positive bone surface (*n* = 8). *P < 0.001; ns, not significant. The data were analyzed using 1-way ANOVA with Tukey’s post hoc tests. Scale bars: 50 μm. b, bone; M1, maxillary first molar; M2, maxillary second molar.

Histological analysis of the alveolar bone under the furcation area revealed that the marrow cavity was enlarged in the T2D group but markedly reduced in the Scl-Ab groups (Figure 4, A–C). In the T2D+Scl-Ab group, the bone and marrow space proportions were restored to those of the control group (Figure 4, A and D), demonstrating that Scl-Ab rescued the T2D-induced bone loss. Scl-Ab treatment strongly promoted bone accumulation in the vicinity of the bone marrow, as shown by Pentachrome staining (Figure 4, E–H). Alkaline phosphatase (ALP) staining confirmed the elevated bone formation activity around the bone marrow region in groups treated with Scl-Ab (Figure 4, I–L). We found fewer bone-lining cells and osteoprogenitors in the bone marrow in the T2D group (Figure 4, M and N, quantified in Figure 4Q). However, in the Scl-Ab groups, more RUNX2^+^ cells were found on the bone surface. Their morphology was cuboidal rather than flat, suggesting they were active bone-forming cells (Figure 4O). In the T2D+Scl-Ab group, a large number of RUNX2^+^ cells were also found on the bone surface (Figure 4P), demonstrating that Scl-Ab treatment promotes bone accumulation under T2D conditions. Consistent with the notion that Scl-Ab suppresses the function of sclerostin, Wnt signaling was elevated in bones from mice treated with Scl-Ab, as evidenced by the presence of active β-catenin^+^ cells (Figure 4, R–U, quantified in Figure 4V).

A similar increase in the number of osteogenic RUNX2^+^ cells was found on the edentulous ridge surface after Scl-Ab treatment (Supplemental Figure 2, A–H). Previously, we reported that mice carrying a null mutation in *Sost (Sost^–/–^* mice)*,* the gene encoding sclerostin, exhibited a thicker edentulous ridge and midfacial hypoplasia (17). In the current study, Scl-Ab treatment increased the thickness of the edentulous ridge (Supplemental Figure 3, A and B) but had no notable effect on the premaxillary suture (Supplemental Figure 3, C–H) or facial length (Supplemental Figure 3, I and J). *Sost^–/–^* mice also displayed enlarged mandibles (17), mimicking the mandibular overgrowth in patients with van Buchem disease and sclerosteosis (17). In the current study, 4 weeks of Scl-Ab treatment resulted in slightly thicker cortical bone (Supplemental Figure 4, A–D). Collectively, our data demonstrate that Scl-Ab exerts a strong anabolic effect on alveolar bone.

### Treatment with Scl-Ab decreases bone resorption.

**Figure 5:**
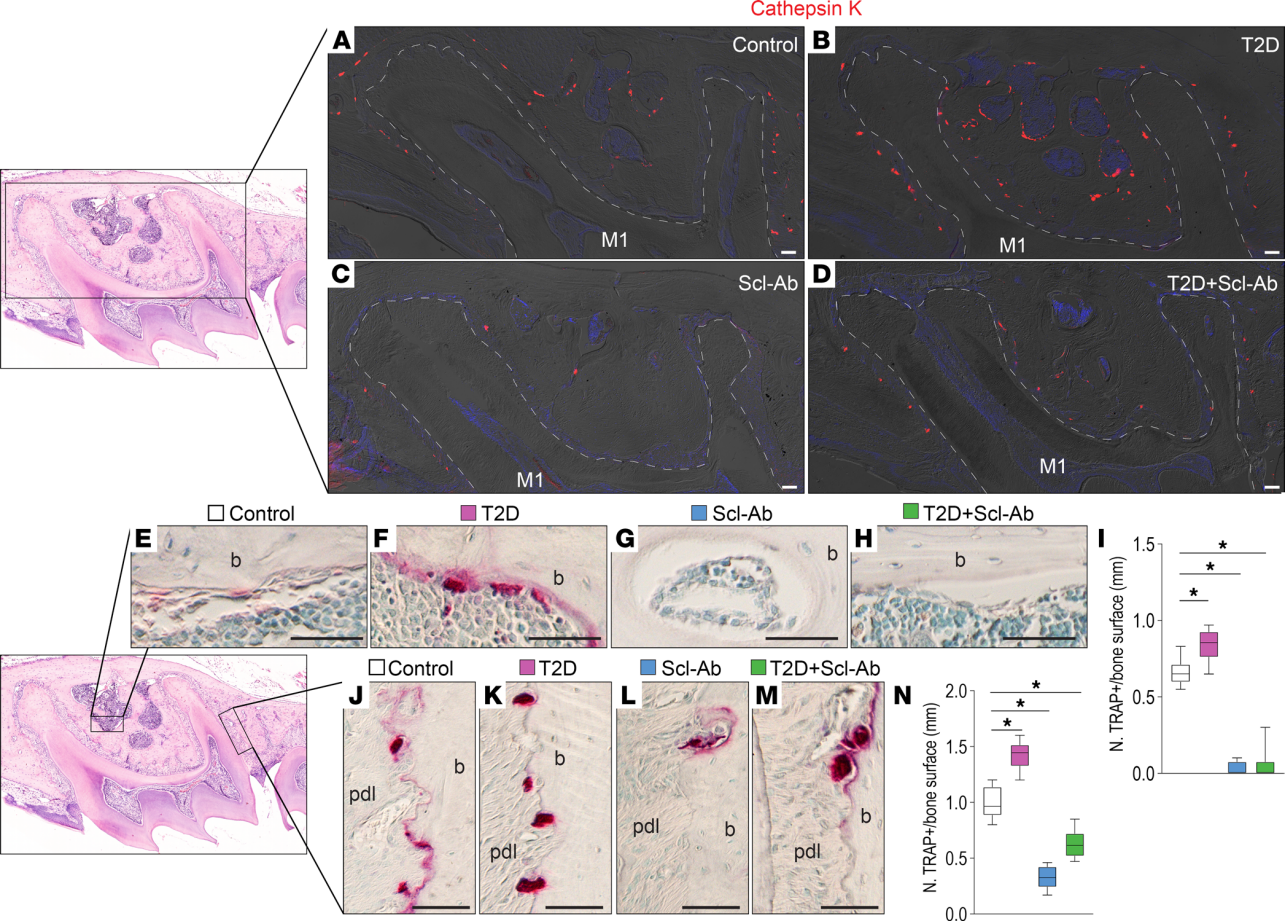
Treatment with Scl-Ab decreases bone resorption. (**A**) In the maxillary first molar area, (**B**–**E**) immunostaining of cathepsin K shows osteoclasts. Dashed lines indicate the shape of the roots. (**F**) In the maxillary first molar bone marrow area, (**G**–**J**) TRAP staining shows bone resorption. (**K**) Quantification of TRAP^+^ cells in the bone marrow area (*n* = 8). *P < 0.01. (**F**) On the mesial sides of the periodontal bone surface, (**L**–**O**) TRAP staining shows bone resorption. (**P**) Quantification of TRAP+ cells on the periodontal bone surface (*n* = 8). *P < 0.0001. The data were analyzed using 1-way ANOVA with Tukey’s post hoc tests. Scale bar: 50 μm. M1, maxillary first molar; pdl, periodontal ligament; b, bone.

To further investigate the effects of Scl-Ab treatment on bone remodeling, we evaluated bone resorption activity in the alveolar bone surrounding the maxillary molar 1 (Figure 5A, boxed area) across different groups. Cathepsin K staining demonstrated an increase in osteoclast numbers in T2D mice, which was reduced in both the bone marrow cavity and PDL area with Scl-Ab treatment (Figure 5, B–E). Tartrate-resistant acid phosphatase (TRAP) staining confirmed an increased number of osteoclasts on the bone facing the marrow of T2D mice (Figure 5F, boxed area). Treatment with Scl-Ab markedly decreased TRAP^+^ cells in both control and T2D mice (Figure 5, G–J, quantified in Figure 5K). Similarly, an elevated number of TRAP^+^ cells was found on bone surfaces facing the PDL in T2D mice (Figure 5F, boxed area), which was markedly reduced after Scl-Ab treatment in both control and T2D mice (Figure 5, L–O, quantified in Figure 5P). Taken together, these findings indicate that Scl-Ab promotes bone formation while inhibiting bone resorption, resulting in rapid bone accumulation.

### Scl-Ab treatment promotes PDL cell proliferation.

**Figure 6:**
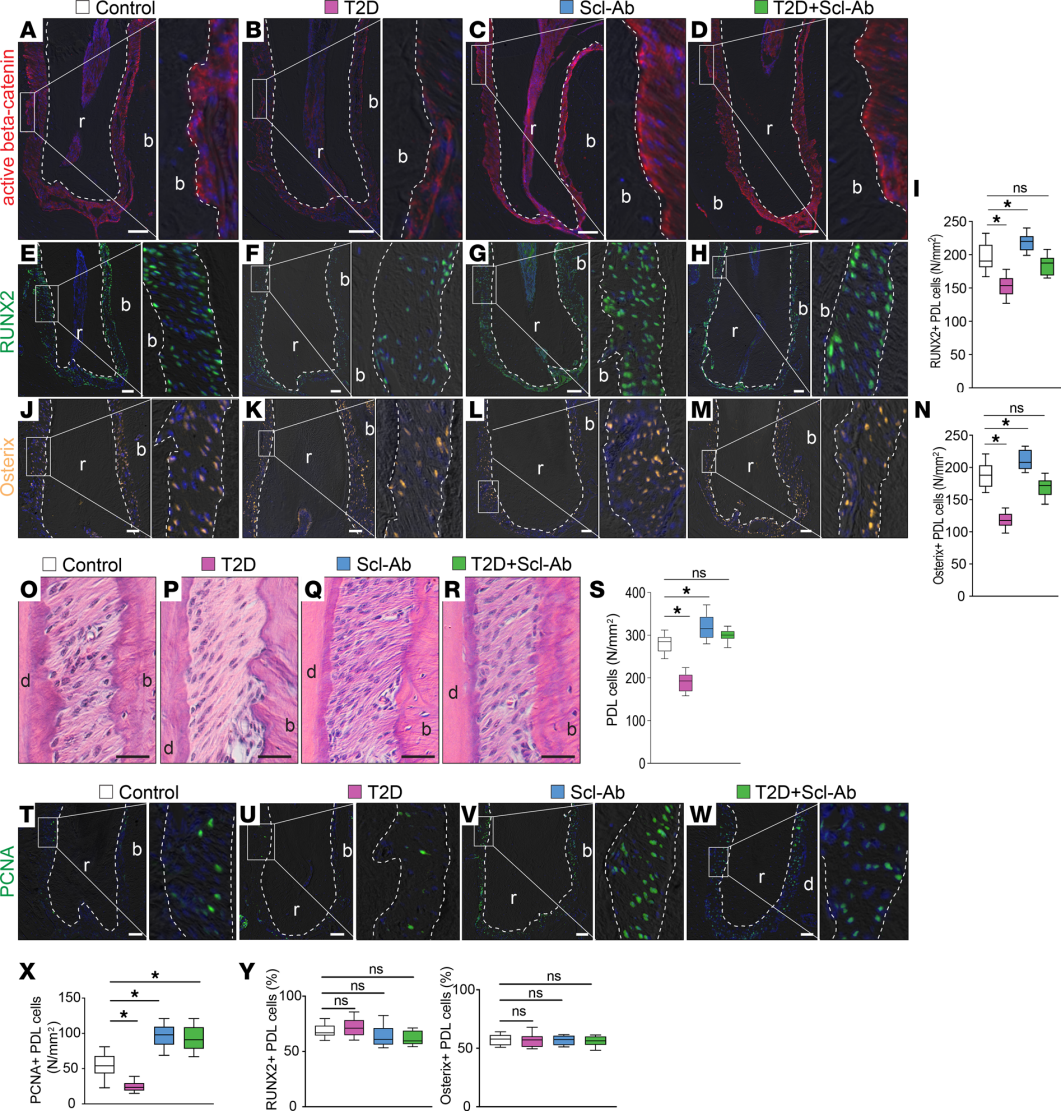
Scl-Ab treatment promotes PDL cell proliferation. (**A**–**D**) Immunostaining of active β-catenin. (**E**–**H**) Immunostaining of RUNX2. (**I**) Quantification of RUNX2^+^ cells in the PDL (*n* = 8). *P < 0.05; ns, not significant. (**J**–**M**) Immunostaining of osterix. (**N**) Quantification of osterix^+^ cells in the PDL (*n* = 8). *P < 0.05. (**O**–**R**) H&E staining shows the PDL. (**S**) Quantification of PDL cells (*n* = 8). *P < 0.0001. (**T**–**W**) Immunostaining of PCNA. (**X**) Quantification of PCNA^+^ cells (*n* = 8). *P < 0.01. (**Y** and **Z**) Quantification of the percentages of (**Y**) RUNX2^+^ and (**Z**) osterix^+^ PDL cells (*n* = 8). Dotted lines indicate the demarcation between the PDL and alveolar bone or dentin/cementum. The data were analyzed using 1-way ANOVA with Tukey’s post hoc tests. Scale bar: 50 μm. r, roots; b, bone.

We next investigated the specific effects of Scl-Ab treatment on bone remodeling in the PDL area, where PDL cells actively participate in alveolar bone remodeling (18, 19). We found that active β-catenin expression was increased in the PDL following Scl-Ab treatment (Figure 6, A–D). Numbers of RUNX2^+^ cells (Figure 6, E–H, quantified in Figure 6I) and osterix^+^ cells (Figure 6, J–M, quantified in Figure 6N) decreased in the T2D PDLs compared with those in controls, but Scl-Ab treatment increased both cell populations in the PDL.

In T2D mice, the number of PDL cells was also reduced (Figure 6, O and P, quantified in Figure 6S). Scl-Ab treatment increased PDL cells and completely rescued this loss in T2D mice (Figure 6, O–R, quantified in Figure 6S). Using PCNA as a cell proliferation marker, we found that cell proliferation was reduced in the T2D group but increased after Scl-Ab treatment (Figure 6, T–W, quantified in Figure 6X). To account for differences in total PDL cells across groups, we calculated the percentage of osterix^+^ and RUNX2^+^ cells (Figure 6, Y and Z). The results demonstrated that, although the absolute numbers of osterix^+^ and RUNX2^+^ cells varied, their proportions in PDL cells were similar across the 4 groups. In conclusion, blocking sclerostin with antibodies boosted Wnt signaling in the PDL, which preferentially promoted proliferation of PDL cells rather than differentiating cells along the osteogenic lineage.

### Scl-Ab therapy stimulates PDL repair.

**Figure 7:**
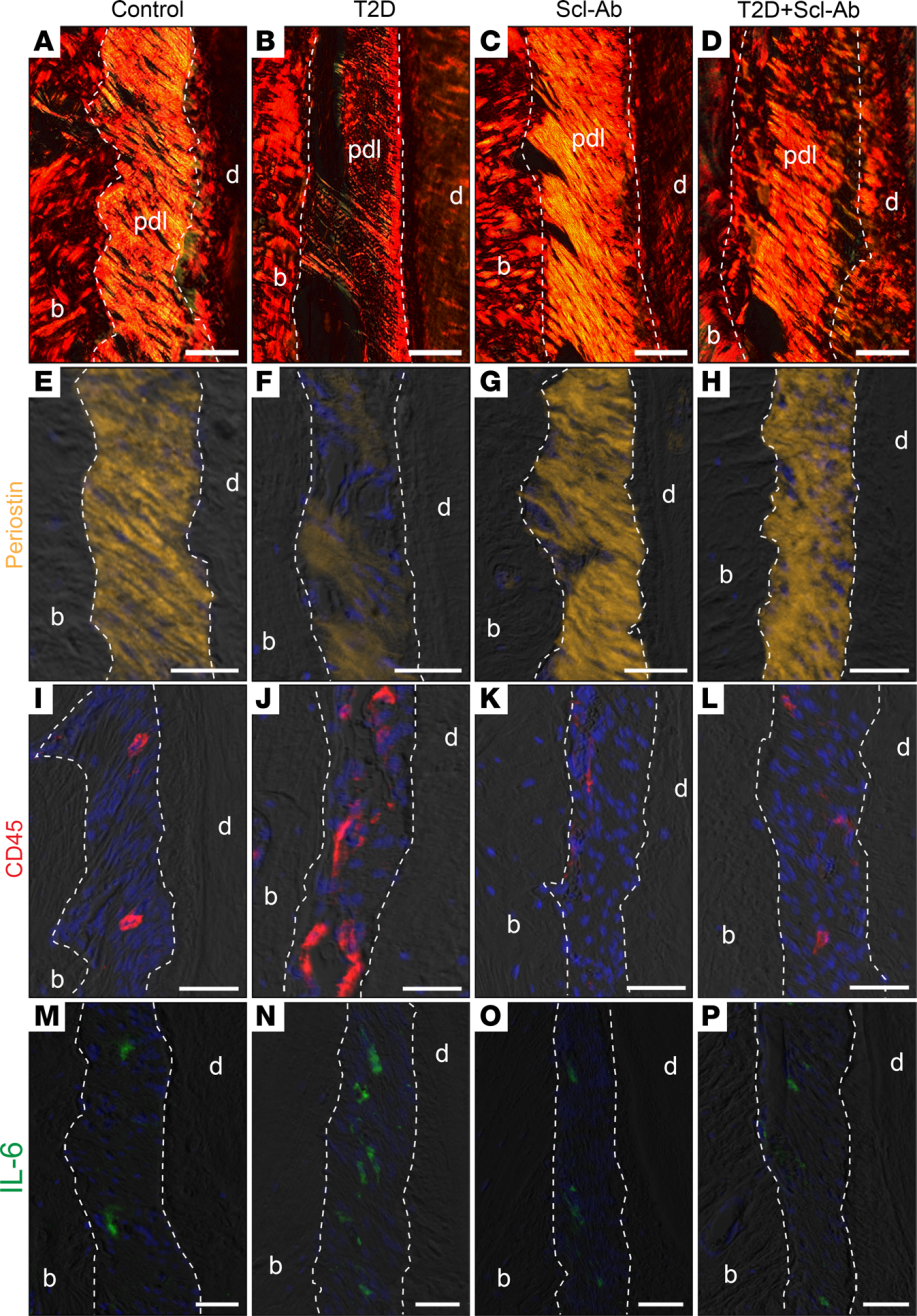
Scl-Ab therapy stimulates PDL repair. (**A**–**D**) Picrosirius red staining, viewed under polarized light. (**E**–**H**) Immunostaining of periostin. (**I**–**L**) Immunostaining of CD45. (**M**–**P**) Immunostaining of IL-6. Dotted lines indicate the demarcation between the PDL and either alveolar bone or dentin/cementum. Scale bar: 50 μm. pdl, periodontal ligament; b, bone; d, dentin.

PDL, which connects the tooth to the alveolar bone, structurally comprises approximately 60% connective tissue fibers by volume. The PDL also contains various cellular components, such as fibroblasts, epithelial cells, osteoblastic cells, and mesenchymal stem cells (20, 21). We investigated the integrity of the PDL and found that the PDL of control mice was covered with abundant and well-aligned collagen fibers, as evidenced by Picrosirius red staining (Figure 7A). In contrast, the number of fibers covering the PDL in T2D mice was substantially reduced (Figure 7B). Treatment of control mice with Scl-Ab had a minor affect on PDL fibers (Figure 7C); however, it markedly increased the number of fibers in the PDL of T2D mice (Figure 7D). Moreover, the expression of periostin, a critical protein for PDL integrity and function (22), was notably decreased in T2D mice compared with control mice, and periostin expression was well preserved in T2D mice treated with Scl-Ab (Figure 7, E–H).

Patients with T2D exhibit inflammation in the periodontal tissues (23, 24). Consistently, we found accumulation of CD45^+^ immune cells in the PDL of T2D mice, which were substantially reduced with Scl-Ab treatment (Figure 7, I–L). Additionally, the PDL of T2D mice contained more cells expressing the proinflammatory cytokine IL-6 compared with the PDL of control mice, and Scl-Ab treatment decreased the abundance of IL-6-expressing cells to levels similar to control mice (Figure 7, M–P). These data indicate that Scl-Ab suppresses the elevated T2D-induced inflammation in the PDL, providing a favorable environment for PDL recovery. In summary, Scl-Ab stimulates PDL repair by promoting PDL cell proliferation and reducing inflammation in the PDL.

Because we observed gingival inflammation in the T2D mice (Figure 1), we investigated whether Scl-Ab treatment could mitigate this inflammation (Supplemental Figure 5, A–D). Scl-Ab treatment reduced the increased accumulation of CD45^+^ immune cells observed in T2D mice; however, levels remained higher compared with those in the control group (Supplemental Figure 5, E–I). Neutrophil levels remained elevated in both T2D mice and those treated with Scl-Ab compared with control mice (Supplemental Figure 5, J–N). These findings suggest that, while Scl-Ab partially mitigates gingival inflammation in T2D mice, its effect is limited. Additionally, we examined cementum thickness. T2D did not notably affect cementum thickness in our mouse model. However, Scl-Ab treatment increased cementum thickness in the furcation area (Supplemental Figure 5, O–S).

## Discussion

In this study, we utilized a nongenetic mouse model to mimic human adult-onset T2D. This HFD/STZ-induced T2D model reproduces the effects of diabetes on the bones of the axial and appendicular skeletons (12). However, the periodontal phenotype has not been characterized. We revealed that, similar to patients with diabetes, HFD/STZ-induced T2D mice display periodontal disorders, including severe alveolar bone loss, gingival inflammation, and PDL degeneration.

Consistent with previous evidence from bones of the axial or appendicular skeleton (10), we found elevated expression of sclerostin in the alveolar bone of diabetic mice. The regulation of *Sost* transcription and sclerostin synthesis is complex and has been intensely investigated since the identification of the *Sost* gene through genetic linkage analysis in patients with sclerosteosis and van Buchem disease (7, 25). *Sost* transcription is controlled by a number of regulatory elements in its promoter region as well as by epigenetic mechanisms (reviewed elsewhere, ref. 26). Various transcription factors activated by cytokines, growth factors, or hormones have been implicated in the positive or negative regulation of *Sost/*sclerostin. The regulation of *Sost/*sclerostin in the context of diabetes is not completely understood and likely results from the combined effects of metabolic and hormonal changes induced by the disease in vivo. Notably, osteocytic cell lines cultured in media containing high concentrations of glucose exhibit elevated *Sost* mRNA and sclerostin protein expression (10, 27), strongly suggesting that glucose directly regulates *Sost* gene expression. Further studies are warranted to fully elucidate the mechanisms underlying the changes in *Sost*/sclerostin expression in diabetes.

Considering the dramatic increase in sclerostin-mediated Wnt inhibition in T2D, we explored the Scl-Ab in this T2D mouse model. Our data indicate that Scl-Ab completely reversed the detrimental effects of T2D on alveolar bone. Scl-Ab stimulated osteoprogenitors and suppressed osteoclasts, leading to the rapid formation of alveolar bone. Moreover, we observed PDL repair in the Scl-Ab–treated T2D group, as evidenced by an increase in PDL cell number and PDL fiber density and a decrease in inflammation. Taken together, this study establishes a T2D mouse model with periodontal disease phenotype and demonstrates that Scl-Ab therapy effectively rescues the periodontal detriments of T2D.

### HFD/STZ-induced adult-onset T2D mouse model.

The HFD/STZ-induced adult-onset T2D mouse model offers advantages for studying T2D-associated periodontal disease over commonly used genetic rodent models. While models like *db/db* mice (28–31) and ZDF rats (32–35) have provided insights into disease pathogenesis, they are limited by factors such as cost, life span, early-onset timing, and relevance to polygenic human T2D (36–39). In contrast, the HFD/STZ model better reflects the adult-onset and metabolic characteristics of human T2D (36, 40), making it a valuable tool for studying adult disease timelines and evaluating therapeutic compounds. Our findings define the periodontal disease phenotype in this model, paving the way for future investigations into the mechanisms linking T2D to periodontitis and preclinical testing of therapeutic strategies against T2D-associated periodontal degeneration.

### Scl-Ab and the craniofacial complex.

Our study demonstrates the remarkable efficacy of Scl-Ab in promoting robust alveolar bone regeneration in our T2D mouse model (Figure 3). Notably, our findings reveal complete restoration of T2D-induced alveolar bone loss after only 4 doses of Scl-Ab. Scl-Ab acts by converting bone-lining cells into osteoblasts (14), thereby activating quiescent surfaces for bone formation (Figure 4). While previous studies have shown alveolar bone recovery under experimental periodontitis (41), large alveolar bone injury (42), and tooth extraction and unloading conditions (43), our study demonstrated extensive regeneration in a T2D context.

While genetic deficiency of sclerostin has been associated with mandibular overgrowth and midfacial defects (44), our study suggests that transient Scl-Ab treatment in adulthood may have minimal effects on craniofacial structures (Supplemental Figures 2–4) and cementum (Supplemental Figure 5). However, further investigation into potential craniofacial changes after prolonged Scl-Ab treatment is warranted.

### Scl-Ab and PDL.

Sclerostin inhibition positively affects PDL health. Knockout of periostin, a key matrix protein of PDL, results in an early-onset periodontitis-like phenotype (45). In these periostin-null mice, deleting the *Sost* gene or blocking sclerostin by antibody substantially restored alveolar bone height and, more strikingly, improved the disorganized orientation of the PDL (18). Scl-Ab treatment in dentin matrix protein 1–knockout (Dmp1-knockout) mice also greatly improved alveolar bone and PDL recovery. Although Dmp1 is not expressed in PDL, mice lacking Dmp1 display a severe defect in PDL (46). Thus, PDL degeneration may be secondary to the alveolar bone defect, and the PDL protective effect of Scl-Ab may be an indirect result of alveolar bone regeneration. A recent study using lineage tracing demonstrated that the activity of the PDL stem cells dramatically increased in *Sost^–/–^* mice (47), suggesting that sclerostin directly affects PDL cells. Our study shows that Scl-Ab treatment stimulated PDL cell proliferation (Figure 6), which is necessary for PDL repair. We propose that Scl-Ab PDL regeneration involves both enhanced alveolar bone formation and activated PDL cell proliferation, stimulating Sharpey’s fiber restoration.

Given that PDL and alveolar bone impairments contribute to most cases of adult tooth loss, the capacity of Scl-Ab therapy to regenerate both bone and PDL underscores its potential as a promising approach for periodontal tissue repair. Continued exploration of the molecular mechanisms underlying sclerostin inhibition will further inform the development of therapeutic strategies against periodontitis and tooth loss.

### Conclusion.

Using an adult-onset mouse model of established T2D, we demonstrated severe alveolar bone loss with gingival inflammation and PDL degeneration. Treatment with Scl-Ab completely reversed these deleterious effects of T2D on the periodontium, restored bone mass by promoting osteoblast activity while suppressing osteoclasts, and stimulated periodontium regeneration while minimally affecting other craniofacial structures.

## Methods

### Sex as a biological variable.

In this study, we exclusively used male mice owing to previous findings indicating that males exhibit a more pronounced response to a HFD and STZ treatment compared with females (48–50). While our findings provide insights into T2D-induced periodontitis and Scl-Ab as a treatment plan in male mice, future studies should investigate whether these findings extend to female mice.

### Mouse model.

The mice were housed in a temperature-controlled environment with 12-hour light/dark cycles and had ad libitum access to food and water. To induce T2D, 12-week-old C57BL/6J male mice (The Jackson Laboratory) were randomly assigned to 2 groups. One group was fed a LFD comprising 10% kcal% fat (D12450J, Research Diet Inc.), while the other group was fed a HFD comprising 60% kcal% fat (D12492, Research Diet Inc.). Four weeks later, the HFD group received 5 daily injections of STZ (45 mg/kg dissolved in vehicle, MilliporeSigma), whereas the LFD group received vehicle (50 mM sodium citrate buffer, pH 4.5). Eight weeks later, T2D was fully developed (fasted blood glucose, >250 mg/dL), and half of the mice from each group received Scl-Ab treatment (100 mg/kg romosozumab) once a week for 4 weeks (12). The control and T2D groups were administered a vehicle (saline) at the same time. All mice were sacrificed at 28 weeks of age for periodontal analysis (Figure 1A).

### Micro-CT analysis.

The heads were scanned using a Skyscan 1176 (Bruker) with the following conditions: X-ray energy of 59 kV, pixel size of 9 μm, and a rotation of 0.3 degrees. The scanned data were reconstructed as previously reported (51). To calculate bone volume and bone volume fraction, the alveolar bone in the furcation area of the maxillary first molars or mandibular first molars was measured by CTAn (Bruker). The regions of interest were previously described (17, 52). The CEJ-ABC distance underneath the distopalatal cusp and distobuccal cusp of the maxillary first molar was measured 3 times, and the average was taken. PDL width was measured by CTAn using the method we described before (53).

### Sample preparation.

The samples were decalcified with 0.5 M EDTA (pH 7.2) for 5 days. After decalcification, the specimens were dehydrated, cleared in xylene, and infiltrated with a xylene-paraffin mixture, followed by paraffin embedding. Sagittal sections with a thickness of 6 microns were cut and collected on positively charged slides.

### H&E stain, Masson’s trichrome stain, and Pentachrome stain.

The sections were deparaffinized and rehydrated before each staining. For H&E staining, we used a previously described method (54). For Masson’s trichrome staining, the slides were stained with Weigert’s Iron Hematoxylin (26758-01/02, Electron Microscopy Sciences) for 8 minutes, Biebrich Scarlet-Acid Fuchsin (26367-04, Electron Microscopy Sciences) for 6 minutes, Phosphomolybdic acid-phosphotungstic acid (26367-05, Electron Microscopy Sciences) for 15 minutes, and Aniline blue solution (26367-06, Electron Microscopy Sciences) for 1 minute. The slides were then differentiated with 1% acetic acid for 5 minutes. For Pentachrome staining, the slides were sequentially stained with 1% Alcian Blue, Orcein-Verhoeff working solution (prepared with Orcein, alcoholic hematoxylin, ferric chloride, and Lugol’s iodine), Woodstain Scarlet-Acid Fuchsin solution (26385-07, Electron Microscopy Sciences), 5% phosphotungstic acid (26385-09, Electron Microscopy Sciences), and 3% Saffron solution (Sigma-Aldrich). After staining, the slides were then dehydrated through graded ethanol, cleared with xylene, and mounted with Permount mounting media.

### ALP and TRAP stain.

Slides were deparaffinized and treated with a buffer (0.1 M Tris pH 9.0, 50 mM MgCl_2_, 100 mM NaCl, and 0.1% Tween 20) at 37°C for 30 minutes. The slides were then incubated at 37°C with a solution containing BCIP (5-bromo-4-chloro-3-indolyl phosphate; Roche) and NBT (nitro blue tetrazolium chloride; Roche) to develop color. After a brief washing, the slides were counterstained with 0.1% nuclear fast red. The TRAP staining was performed as described before (55), and the slides were counterstained with 0.5% Methyl Green (7114-03-06, Electron Microscopy Sciences) for 15 seconds. The slides for ALP and TRAP staining were dehydrated in a series of ethanol and xylene and then mounted with Permount mounting media.

### Picrosirius red stain.

The slides were stained with 0.1% Sirius Red in saturated picric acid (26357-02, Electron Microscopy Sciences) for an hour, followed by washing with 1% acetic acid. The slides were then dehydrated, mounted, and viewed under polarized light.

### Immunohistochemistry.

Immunohistochemistry staining was performed as described previously (56) using the following primary antibodies: anti-RUNX2 (ab192256, Abcam), active β-catenin (8814, Cell Signaling Technology), anti–cathepsin K (ab300569, Abcam), anti-osterix (ab22552, Abcam), anti-PCNA (24036-1, Proteintech), anti-periostin (19899-1, Proteintech), anti-CD45 (70257, Cell Signaling Technology), anti-Myeloperoxidase (AF3667, R&D Systems), anti–IL-6 (AF406, R&D Systems), anti-sclerostin (AF1589, R&D Systems), and anti-DKK1 (AF1765, R&D Systems). Goat anti-rabbit IgG (H+L) cross-adsorbed antibody (A21244, Invitrogen) and donkey anti-goat IgG (H+L) cross-adsorbed antibody (A21447, Invitrogen) were used as secondary antibodies.

### Quantification.

To quantify Picrosirius red^+^ PDL, Adobe Photoshop (version 22.4) was used. First, we selected the PDL area (between cementum and alveolar bone) using the magnetic lasso tool and recorded the number of pixels as the total pixels. Next, we copied the selected area to a new Photoshop file and used the magic wand tool to select the stained area. We recorded the number of pixels in the selected area as positive pixels. The results are reported as a percentage of positive pixels over the total pixels. To quantify RUNX2^+^osterix^+^ PDL cells, the positive cells were manually counted, and the PDL area was measured using ImageJ (NIH). The same method was used to quantify PDL cells. The results are expressed as the number of positive cells over the total area. The percentages of RUNX2^+^ PDL cells and osterix^+^ PDL cells were also calculated. To quantify TRAP^+^ osteoclasts, positive cells were counted, and the bone surface length was measured by ImageJ (NIH). The results are reported as the number of positive cells over the length of the bone surface. To quantify RUNX2^+^ bone-lining cells, the number of RUNX2^+^ cells along the bone surface was counted and normalized to the bone surface length, which was measured using ImageJ software. For quantification of active β-catenin^+^ bone surface, both the active β-catenin^+^ bone surface and the total bone surface were measured using ImageJ and expressed as a percentage. To measure cementum thickness, the furcation area was selected, and measurements were taken randomly at multiple sites on each slide. The measurements were averaged to obtain the mean cementum thickness. A minimum of 3 stained sections were analyzed for each sample.

### Statistics.

Results are presented as mean ± SD. A 2-tailed Student’s *t* test was used to compare between 2 groups. For comparisons involving more than 2 groups, a 1-way ANOVA followed by Tukey’s post hoc test was employed. Prism 9 (GraphPad Software) was used for all statistical analyses. A *P* value of less than 0.05 was considered significant.

### Study approval.

All animal procedures were conducted with approval of and in accordance with guidelines set by the Institutional Animal Care and Use Committee at the University of Arkansas.

### Data availability.

Values for graphs in the figures and supplemental figures are provided in the Supporting Data Values file.

## Author contributions

HT, XY, and TB designed the research. SF, TZ, NA, SM, DOG, and XY performed data acquisition. HT and XY analyzed the data. XY drafted the manuscript. HT, SF, TZ, NA, SM, DOG, XY, and TB critically revised the manuscript.

## Supplementary Material

Supplemental data

Supporting data values

## Version 1 07/18/2024

In-Press Preview

## Version 2 08/22/2024

Electronic publication
