# Supplementary material for: Sclerostin antibody corrects periodontal disease in type 2 diabetic mice
Source: JCI Insight. 2024 Jul 18;9(16):e181940. doi: 10.1172/jci.insight.181940 (PMC11343605; doi:10.1172/jci.insight.181940)
Supplement: Supplemental data [file jciinsight-9-181940-s128.pdf]

Sclerostin antibody corrects periodontal disease in type 2 diabetic mice

Hakan Turkkahraman[1], Shannan Flanagan[2], Tianli Zhu[3], Nisreen Akel[4], Silvia Marino[4], Dayane Ortega-Gonzalez[2], Xue Yuan[2,5], Teresita M. Bellido[4,6]

[1] Indiana University School of Dentistry, Department of Orthodontics and Oral Facial Genetics, Indianapolis, IN, USA.

[2] Department of Otolaryngology-Head & Neck Surgery, School of Medicine, Indiana University, Indianapolis, IN, USA.

[3] Department of Biomedical Sciences and Comprehensive Care, Indiana University, School of Dentistry, Indianapolis, IN, USA.

[4] Department of Physiology and Cell Biology, University of Arkansas for Medical Sciences, Little Rock, AR, USA.

[5] Indiana Center for Musculoskeletal Health, School of Medicine, Indiana University, Indianapolis, IN, USA.

[6] Central Arkansas Veterans Healthcare System, John L. McClellan Little Rock, AR, USA.

Corresponding authors:

Xue Yuan, PhD, 1160 West Michigan Street, GK305, Indianapolis, IN, 46202. Telephone number:317-274-4921. Email: yuanxue@iu.edu

Teresita Bellido, PhD, West Markham Street, Slot 505, Little Rock, AR, 72205. Telephone number: 317-645-8897. E-mail: TMBellido@uams.edu

Supplemental Figures and Figure Legends

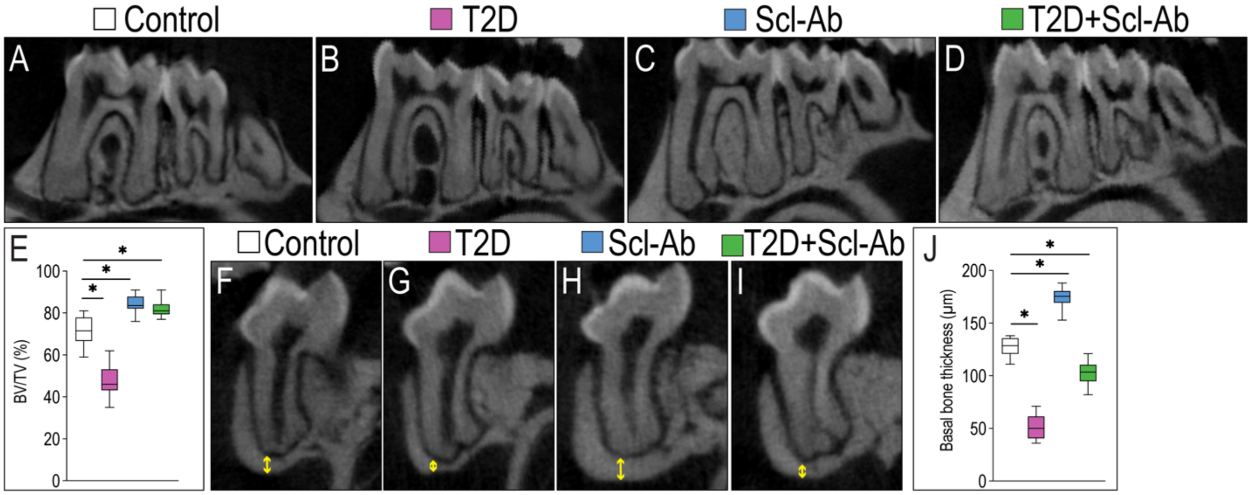

Supplemental Figure 1. Scl-Ab treatment reverses T2D-induced alveolar bone loss.
(A-D) Representative 2-dimensional (2D) µCT sagittal sections through the alveolar bone around mandibular first molars. (E) Quantification of bone volume over tissue volume (BV/TV) (n = 8). P<0.05. (F-I) Representative 2D µCT coronal sections through the alveolar bone around maxillary first molars. Arrows indicate the thickness of the basal bone. (J) Quantification of the thickness of the basal bone (n = 8). P<0.01.

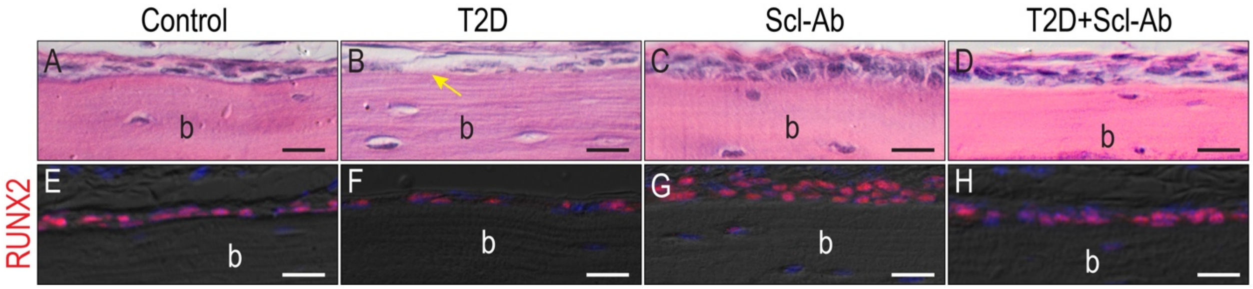

Supplemental Figure 2. Scl-Ab promotes osteoprogenitor formation on the maxillary edentulous periosteal bone surface.

(A-D) H&E staining shows the edentulous maxillary periosteal bone surface. The arrow indicates the bone surface that is not covered by cells. (E-H) Immunostaining of RUNX2. Abbreviation: b, bone. Scale bars: 25 μm.

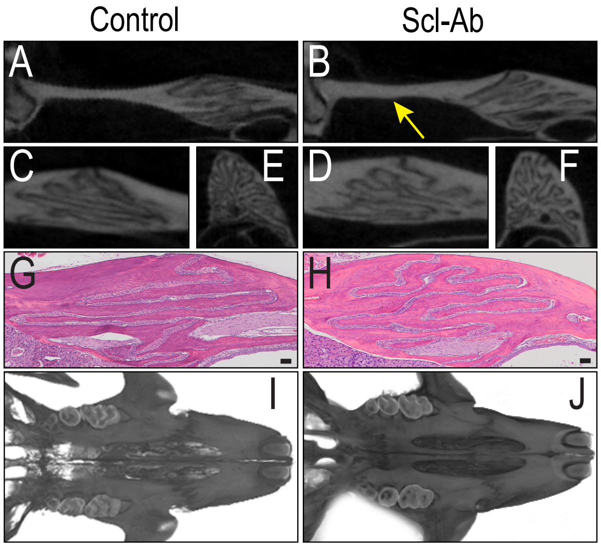

Supplemental Figure 3. Scl-Ab treatment alters the edentulous ridge but not the premaxillary suture.

(A, B) Representative sagittal 2D micro-CT sections of the maxillary molar 1 to premaxillary suture area. The arrow indicates the enlarged edentulous ridge in the Scl-Ab group. (C, D) Enlarged premaxillary suture areas in the Scl-Ab group compared with the control group. (E, F) Representative coronal 2D micro-CT sections of the premaxillary suture. (G, H) Representative H&E-stained histological sagittal sections through the premaxillary suture. (I, J) Representative 3D micro-CT reconstruction images of the skull. Scale bars: 50 μm.

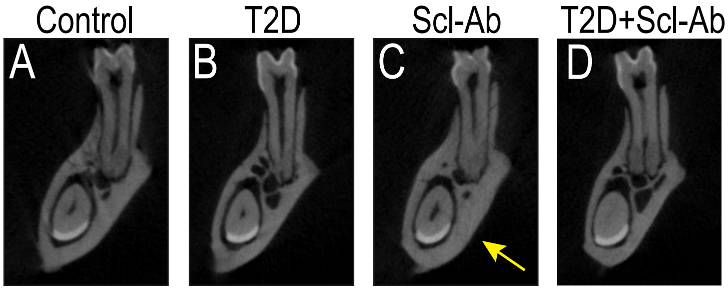

Supplemental Figure 4. Scl-Ab treatment slightly enlarges the mandible.

(A-D) Representative coronal 2D micro-CT sections of mandibles. The arrow indicates increased thickness.

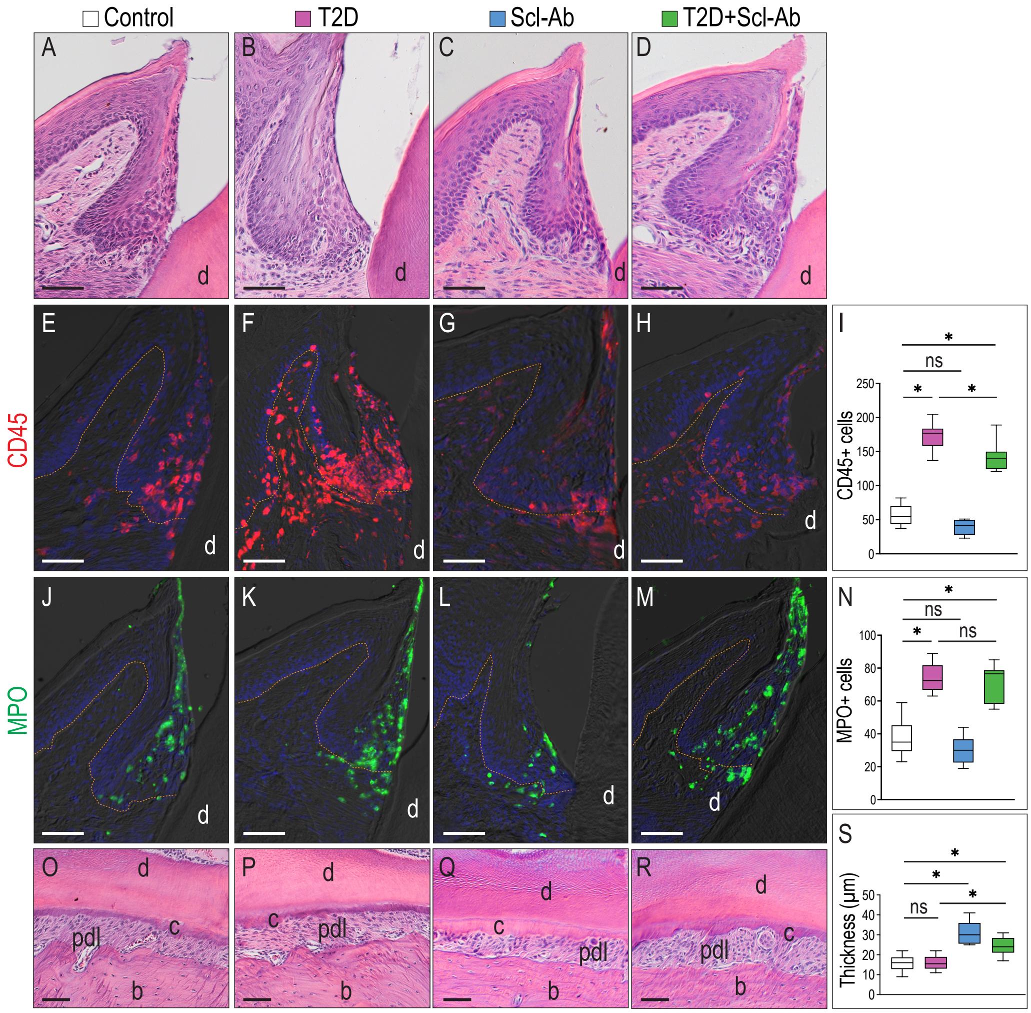

Supplemental Figure 5. Gingival inflammation and cementum thickness change after Scl-Ab treatment.

(A-D) H&E staining shows the gingiva of the first maxillary molar. (E-H) Immunostaining of CD45. (I) Quantification of CD45+ cells. (J-M) Immunostaining of the neutrophil marker myeloperoxidase (MPO). P<0.01. (N) Quantification of MPO+ cells. P<0.0001. (O-R) H&E staining shows cementum in the furcation area. (S) Quantification of cementum in the furcation area (n = 8). P<0.01; ns, not significant. d, dentin; c, cementum; pdl, periodontal ligament; b, bone. Scale bars: 50 μm.
